# Antagonizing FcαR1 (CD89) as treatment in IgA-mediated chronic inflammation and autoimmunity

**DOI:** 10.3389/fimmu.2023.1118539

**Published:** 2023-04-04

**Authors:** Myrthe A. M. van Delft, Esil Aleyd, Richard van der Mast, Niels de Jong, Louis Boon, Peter J. Simons, Marjolein van Egmond

**Affiliations:** ^1^ Molecular Cell Biology and Immunology, Amsterdam University Medical Center (UMC) location Vrije Universiteit Amsterdam, Amsterdam, Netherlands; ^2^ Inflammatory Diseases, Amsterdam Institute for Infection and Immunity, Amsterdam, Netherlands; ^3^ Research and Development, Polpharma Biologics, Utrecht, Netherlands; ^4^ Research and Development, JJP Biologics, Warsaw, Poland; ^5^ Surgery, Amsterdam University Medical Center (UMC) Vrije Universiteit Amsterdam, Amsterdam, Netherlands

**Keywords:** immunoglobulin A, Fc alpha receptor (FcαRI), neutrophils (PMNs), chronic-inflammation (CI), autoimmunity

## Abstract

**Introduction:**

Immunoglobulin A (IgA) is mostly considered as a non-inflammatory regulator at mucosal areas. However, previous work of our group showed that IgA can also be involved in disease pathology, because it provides a potent stimulus to activate neutrophils after crosslinking of surface CD89 (FcaRI), resulting in chronic inflammation and tissue damage. IgA (auto)antibodies and neutrophils are key players in various diseases, including blistering skin diseases and rheumatoid arthritis. Therefore, we generated an array of anti-CD89 monoclonal antibodies (mAbs) for therapeutic targeting of CD89. The biological activity of newly developed anti-human CD89 mAbs and their potential therapeutic capacity were investigated.

**Methods:**

Human neutrophils were isolated from heparinized healthy donor blood. The ability of anti-CD89 mAbs to bind human neutrophils was investigated by flow cytometry. Furthermore, the capacity of these anti-CD89 mAbs to inhibit IgA-mediated phagocytosis, neutrophil extracellular trap (NET) release and migration was studied. To this end, neutrophils were pre-incubated with/without anti-CD89 mAbs after which they were stimulated with IgA-coated beads. The amount of phagocytosed beads, NET release and migrated neutrophils were subsequently analysed. In parallel, chemoattractant leukotriene B4 and lactoferrin (as a measure for degranulation) release were determined. Finally, the therapeutic potential of our prototypic anti-CD89 mAb clone 10E7 was in vivo tested in anti-mouse collagen XVII human IgA-treated transgenic CD89 mice, a preclinical model for autoimmune linear IgA bullous disease (LABD).

**Results:**

Our results show that all generated anti-CD89 mAbs bound surface CD89 on neutrophils. Although these anti-CD89 mAbs bind to different epitopes on EC1 of CD89, they all have the capacity to inhibit IgA-mediated phagocytosis, neutrophil extracellular trap (NET) release and neutrophil migration. Moreover, IgA mediated leukotriene B4 and lactoferrin release are decreased in supernatant from anti-CD89 mAbs-treated neutrophils. Finally, anti-CD89 mAb clone 10E7, that was selected based on its selective binding profile on tissue micro arrays, reduced anti-mouse collagen XVII hIgA-induced neutrophil influx in an in vivo linear IgA bullous disease (LABD) mice model.

**Conclusion:**

This study clearly indicates that our newly developed anti-CD89 mAbs inhibited IgA-induced neutrophil activation and reduced anti-autoantigen IgA-induced neutrophil influx in vivo, supporting further clinical development for the treatment of LABD.

## Introduction

Immunoglobulin (Ig) A is the predominant antibody present at mucosal areas, and second prevalent in serum. Moreover, IgA is the highest produced antibody class of the human body either as monomers or dimeric antibody (1, 2). Monomeric IgA is mainly produced by plasma cells in the bone marrow, whereas dimeric IgA is typically produced by local plasma cells at mucosal surfaces (1, 3, 4). Dimeric IgA is transported through epithelial cells after covalent binding to the poly-Ig-receptor which cleavage leads to the release of secretory IgA at the luminal site.

IgA is generally considered as a non-inflammatory regulator at mucosal areas by neutralizing microorganisms in the lamina propria, in epithelial cells and in the lumen (1, 3, 5). Previously, it was assumed that IgA does not potently trigger pro-inflammatory responses, but more recently it has become clear that that IgA is a potent stimulus to activate e.g. neutrophils, osteoclasts or CD103^+^ dendritic cells after crosslinking of their surface IgA Fc receptor (FcαRI, CD89) (6–9). In addition, it has been shown that anti-cancer (bispecific) antibodies targeting CD89 induce superior killing of tumor cells by neutrophils (10, 11).

CD89 is a member of the Fc receptor immunoglobulin superfamily (1), expressed on cells of the myeloid lineage like neutrophils, eosinophils, monocytes macrophages, and platelets (1, 12, 13). Recently, it was shown that CD89 is also expressed on osteoclasts (9). Cross-linking of CD89 by IgA immune-complexes or IgA-opsonized pathogens induces multiple processes in neutrophils, such as phagocytosis and production of reactive oxygen species, inflammatory mediators, cytokines (e.g. interleukin -8) and chemoattractants (mainly leukotriene B4 (LTB_4_)) (6–8, 14). Moreover, uptake of IgA complexes induces release of neutrophil extracellular traps (NETs) (6). We anticipate that this activation is immune-protective when directed against pathogens (15). However, when IgA antibodies are directed to autoantigens, excessive activation of neutrophils by IgA complexes can occur, which strongly contributes to chronic inflammation and tissue damage (16). In such scenario, autoantigen-specific IgA antibodies that have bound to their autoantigens may cross-link CD89, which will subsequently result in neutrophil recruitment *via* LTB4 release (7) and in massive neutrophilic activation.

IgA (auto)antibodies are present in various diseases, including Linear IgA Bullous Disease (LABD), Dermatitis Herpetiformis (DH), and Rheumatoid Arthritis (RA) (reviewed in (1)). LABD patients have IgA autoantibodies and massive neutrophil accumulation in the skin. IgA autoantibodies in this disease are directed against collagen type XVII, which is an important adhesion molecule in the dermo-epidermal junction (17). Neutrophil activation in LABD is likely the direct result of CD89 activation by IgA autoantibodies, which leads to skin damage and blister formation (16, 18). DH patients have IgA autoantibodies against tissue transglutaminase, which cross-react with epidermal transglutaminase, resulting in IgA deposits and infiltration of neutrophils in the dermal papillae (19). RA is a chronic autoimmune disease predominantly affecting synovial joints (20, 21). The presence of autoantibodies is a hallmark of RA and may result in immune complexes formation in the joint, leading to the attraction of immune cells. Well known autoantibodies in RA are IgM rheumatoid factor (RF), IgG anti-citrullinated protein s (ACPA) and IgG anti-carbamylated protein (CarP) antibodies (22, 23). Interestingly, high IgA ACPA and IgA RF titers correlate with a more severe disease outcome and may be used as a predictive biomarker for disease progression (24–27). Blocking the interaction of IgA RF and macrophage CD89 resulted in less TNF-α secretion (28). Interestingly, neutrophils are also abundantly present in synovial fluid of affected joints (29, 30), and blocking the CD89-IgA interaction on neutrophils resulted in reduced NETs release after stimulation with IgA-containing immune complexes that were isolated from synovial fluid of RA patients (31). Moreover, stimulation of osteoclasts with these complexes resulted in IL8 and IL6 release by osteoclasts (9). Overall, this shows that IgA autoantibodies, beyond being a biomarker for disease severity, induce potent pro-inflammatory functions by neutrophils, macrophages, and osteoclasts, thereby contributing to inflammation and erosive disease.

We have therefore developed antagonistic anti-CD89 antibodies for therapeutic targeting to resolve inflammation and ameliorate disease symptoms. Our newly developed anti-CD89 mAbs inhibited IgA-induced neutrophil activation, have no inherent agonistic activity and bind to EC1 of CD89. Moreover, our lead anti-CD89 mAb clone 10E7, has no unexpected cross reactivity to other human tissues and reduced auto-IgA-induced neutrophil influx in the *in vivo* LABD model.

## Materials and methods

### Mice

Transgenic mice (BALB/c background) expressing human CD89 (FcαR1) (32) and knock-in for human IgA (33) were bred and housed at the animal facility of the VU University (Amsterdam, The Netherlands) under standard conditions. Experimental animals were littermates and cohoused. All animal experiments were performed according to the institutional guidelines and procedures. The animal ethical committee of the VUmc approved all animal experiments.

### Isolation of human PMNs from healthy donors

PMNs were isolated from heparinized peripheral healthy donor blood as described before (31). Briefly, cells were separated using lymphoprep (Alere Technologies) density gradient centrifugation after which erythrocytes were lysed using ammonium chloride buffer (155 mM NH_4_Cl (Merck), 10 mM KHCO_3_ (S.T. Baker) and 0.11 mM EDTA (Sigma). PMNs were washed with phosphate buffered saline (PBS; Fresenius Kabi) and resuspended in RPMI1640 (Gibco) supplemented with glutamine (Gibco), penicillin-streptomycin (Gibco) and fetal calf serum (FCS; Biowest). For NET-release assays, 1% heat-inactivated FCS was added (1% RPMI++), and for the other assays, 10% heat-inactivated FCS was added (10% RPMI++). Isolated PMNs were allowed to rest for 30-60 minutes at 37°C before starting experiments. All donors gave informed consent for collecting blood according to the guidelines of the Medical Ethical Committee of the VU University Medical Center (The Netherlands).

### Generation of blocking mouse anti-human CD89 monoclonal antibodies

Hybridomas were established from splenocytes obtained from BALB/c mice immunized with recombinant extracellular human CD89 (Sino Biological) and membrane-bound CD89 in HEK293F cell lysates (see *Domain and epitope mapping* below). Hybridomas producing anti-CD89 mAbs were screened using conventional methods, like ELISA using solid-phase recombinant human CD89 and flow cytometry using human CD89 expressing HEK293F cells as targets, respectively. The blocking potency of anti-CD89 mAbs was evaluated using an in-house ELISA in which serum-derived human IgA (Bethyl Laboratories) binding to solid-phase recombinant human CD89 was measured. Seven hybridomas, which inhibited the binding of human IgA to human CD89 (data not shown), were expanded, and anti-CD89 mAbs (mouse IgG_1_) were purified from collected supernatants with protein A, and subsequently used for experiments.

### Binding of anti-CD89 monoclonal antibodies to PMNs

10^5^ cells were added per well in 96-wells round bottom plates (Greiner bio-one, Cellstar) and stained with 10 µg/ml mouse anti-CD89 mAbs or a mouse IgG_1_ negative isotype control (Biolegend). Subsequently, PE-labeled goat-anti-mouse IgG antibody (Jackson) was added. After final washings, cells were fixed in PBS/0.1% bovine serum albumin (BSA; Fitzgerald)/2%formaldehyde (37%; Sigma). Binding of anti-CD89 antibodies was determined with flow cytometry (FACS Cyan, Beckman Coulter), and expressed as signal-to-noise (S/N) ratios, ie, dividing measured geometric fluorescence intensity of anti-CD89 antibody by measured geometric fluorescence intensity of mouse IgG1 isotype control.

### Coating of beads

Latex beads (carboxylate-modified polystyrene, non-fluorescent (0.9 μM) or green fluorescent (1.0 μM); Sigma-Aldrich) were coated with BSA (Akron) or purified serum IgA (MP Biomedicals) as described previously (6). Shortly, beads were resuspended in 2-(N-morpholino) ethanesulfonic (Sigma) buffer with 2 mg/ml BSA (Akron) or serum IgA (MP Biomedicals) in the presence of N-(3-Dimethylaminopropyl)-N’-acid ethylcarbodimide hydrochlorid (Sigma-Aldrich) and incubated O/N at room temperature (RT) (overhead shaker). After washing, beads were resuspended in PBS/0.1%BSA.

For migration experiments cyanogen Bromide-Activated Sepharose beads (4B from Amersham; GE healthcare) were coated with BSA or IgA as described (7, 16). Briefly, 100 mg beads were washed in 1 mM HCl and then resuspended in 0.1M NaHCO3 (Merck)/0.5M NaCl (Fluka) (pH 8.3). 300 µg BSA (Akron) or IgA (MP Biomedicals) was added to the beads and incubated O/N at 4°C. Beads were washed with 0.1 M NaHCO3/0.5M NaCl (pH 8.3) and incubated with 0.5 ml 0.1M Tris (Merck)/HCl (Sigma)/0.5M NaCl (pH 8.0) for 2 hours (h) at RT. Subsequently beads were washed with alternating 0.1 M NaAc (Sigma)/0.5M NaCl (pH 4.0) and 0.1 M Tris/HCl/0.5M NaCl (pH 8.0) for 3 cycli and resuspended in PBS/20% etOH (for storage) or 1/10%RPMI++ (for use).

### Phagocytosis assay

Isolated PMNs were resuspended in 10%RPMI++. 2x10^5^ cells were seeded per well in 96-wells round bottom plates (Greiner bio-one, Cellstar) and incubated with 20 µg/ml anti-CD89 mAbs or a mouse IgG_1_ isotype antibody as negative control (Biolegend) for 20 minutes (min) at 4°C. Green fluorescent latex beads coated with BSA or IgA were incubated with the cells for 30 min at 37°C a cell-to-bead ratio of 1:60. After washing, fluorescence intensity was measured with flow cytometry (BD FACS Calibur, BD bioscience) and phagocytic index was calculated as the percentage of cells that phagocytized, multiplied by the mean fluorescent intensity of bead-positive cells (6).

### Quantitative fluorimetric analysis of NET- release, 2-D migration, and human IgA ligand binding

The release of neutrophil extracellular traps (NETs) was analyzed as previous described (6). 10^5^ isolated PMN cells were seeded per well in 96-wells round bottom plates (Greiner bio-one, Cellstar) and incubated with 20 µg/ml anti-CD89 mAbs or a mouse IgG_1_ isotype antibody as negative control (Biolegend) for 20 min at 4°C. Cells were incubated with non-fluorescent latex beads coated with BSA or IgA for 30 min at 37°C at a cell-to-bead ratio of 1:300. After washing, PMNs were resuspended in 1%RPMI++ and transferred to a black 96-wells plate (Greiner). 10 nM Phorbol myristate acetate (PMA; Sigma) was added to the positive control wells and 100 µg/ml DNAse 1 (Roche) to the negative control wells. PMNs were incubated for 3 h at 37°C after which extracellular DNA was detected by adding 2.5 µM SYTOX Green (Invitrogen Life Technologies). Optical density was measured with a fluorimeter (FLUOstar/POLARstar BMG Labtech GmbH) at 480 nm excitation, 520 nm emission.

For 2-D migration isolated PMNs were resuspended in 10%RPMI++ medium in a concentration of 4x10^6^ cells/ml (16). Cells were labeled with 1 µM Calcein AM (green fluorescence; ThermoFisher Scientific) for 30 min at 37°C, after which 2.5x10^5^ cells were seeded per well in 96-wells flat bottom plates (Greiner bio-one, Cellstar) to form a monolayer, and incubated with 20 µg/ml anti-CD89 mAbs or a mouse IgG_1_ isotype antibody as negative control (Biolegend) for 20 min at 4°C. IgA and BSA coated sepharose beads were carefully added on top of the monolayer PMNs, and incubated for 40 min at 37°C after which supernatant was removed and beads were washed with PBS to remove unbound cells. PMNs bound to the beads were lysed using HTAB (Hexadecyltrimethylammonium bromide, containing 1 g/L Tween-20 (Sigma), 2 g/L CTAB (Sigma), 2 g/L BSA (Fitzgerald), 7.44 g/L Na-EDTA (Sigma)). Optical density was measured with a fluorimeter (FLUOstar/POLARstar BMG Labtech GmbH) at 485 nm excitation, 520nm emission. The amount of LTB_4_ in the supernatant was measured by ELISA (see below).

For ligand binding assays, 96-wells Maxisorp™ plates (Nunc Maxisorp) were coated with 10 µg/ml human IgA (MP Biomedicals) or BSA (Akron). Isolated PMNs were labeled with 1 µM Calcein AM (green fluorescence; ThermoFisher Scientific). After that, 2x10^5^ cells were seeded per well to 96-wells flat bottom plates (Greiner bio-one, Cellstar) and incubated with 20 µg/ml anti-CD89 mAbs or a mouse IgG_1_ isotype antibody as negative control (Biolegend) for 20 min at 4°C. Subsequently, cells were transferred to coated Maxisorp™ plates and incubated for 30 min at 37°C, after which supernatant was collected and stored at -20°C. Plate bound cells were washed with PBS and lysed using HTAB. Optical density was measured with a fluorimeter (FLUOstar/POLARstar BMG Labtech GmbH) at 485 nm excitation, 520 nm emission. The amount of lactoferrin in the supernatant was measured with ELISA (see below).

Standard curves with known numbers of lysed calcein AM–labeled neutrophils (0 – 3 x 10^5^ cells/well) were used to quantify to numbers 2-D migrated or IgA (ligand)-bound neutrophils.

### Measurement of LTB_4_ and lactoferrin in supernatant of activated PMNs

The amount of LTB_4_ in supernatants from 2-D migration assays was measured with a commercial LTB_4_ ELISA kit (Enzo life sciences) according to manufacturer’s protocol.

Lactoferrin in the supernatants of ligand binding assays was measured with an in-house developed ELISA (31). Briefly, Maxisorp™ plates (Nunc Maxisorp) were coated with 50 µg/ml polyclonal rabbit-anti-human-lactoferrin antibodies (Sigma, L-3262) O/N at 4°C and blocked with PBS/0.005% Tween-20/0.5% BSA 1 h at 37°C. Two times diluted supernatants were added to plates and incubated for 1h at 37°C followed by incubation with alkaline phosphatase-labeled rabbit-anti-human-lactoferrin antibodies (1:2500, MP Biomedicals) for 1h at 37°C. After adding the chromogenic substrate P-nitrophenyl phosphate (Sigma), optical density was measured with a microplate reader (Biorad) at 405 nm. Purified human lactoferrin (Sigma) was used as a standard.

### Domain and epitope mapping of anti-CD89 mAbs

The following human CD89 constructs were generated for domain mapping and transiently expressed (**Table 1**) (1): wild type human CD89 construct, which contained both Ig-like extracellular 1 (EC1) and Ig-like EC2 domains of human CD89 (SEQ 1), and therefore denoted as ‘human EC1-EC2-CD89’ (2). membrane chimeric Ig-like EC1 domain of human CD89 combined with Ig-like EC2 domain of bovine Fcγ2R construct (SEQ 3 and SEQ 4, i.e., combined with bovine transmembrane-intracellular region or with human transmembrane-intracellular region, respectively), and therefore denoted as ‘human EC1-CD89’ (3). membrane chimeric Ig-like EC1 domain of bovine Fcγ2R combined with Ig-like EC2 domain of human CD89 construct (SEQ 7), and therefore denoted as ‘human EC2-CD89’ (4). membrane full-length bovine Fcγ2R construct was also generated, which contained both Ig-like EC1 and Ig-like EC2 domains of bovine Fcγ2R (SEQ 9), and therefore denoted as ‘bovine Fcγ2R’. cDNAs encoding for above-described ‘human EC1-EC2-D89’, ‘human EC1-CD89’, ‘human EC2-CD89’, and ‘bovine Fcγ2R’ constructs were optimized for mammalian expression and synthesized by GENEART, Regensburg, Germany (SEQs 2, 5, 6, 8, and 10, respectively). These cDNAs were subcloned in pcDNA3.1-derived expression plasmids. Detailed information about SEQs is shown in **Supplement Figure S1**.

**Table 1:** Domain and epitope mapping contructs.

Domain mapping	EC1 EC2 TM/IC
human EC1-EC2-CD89	  
human EC1-CD89	 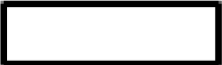 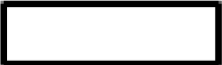
	 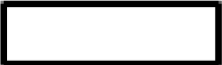 
human EC2-CD89	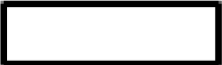  
bovine Fcγ2R	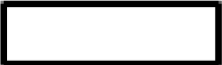 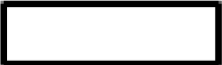 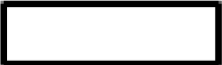
Epitope mapping	
human EC1-CD89	  
™Gln22 – Lys46 human EC1-CD89	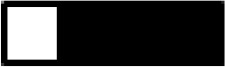  
™Ile47 – Ile71 human EC1-CD89	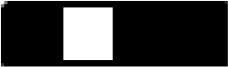  
™Gly72 – Gly96 human EC1-CD89	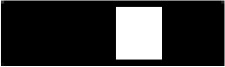  
™Arg97 – Gly121 human EC1-CD89	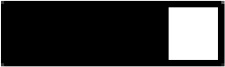  
cynomolgus EC1-CD89	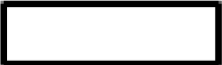 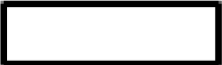 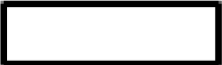

EC, extracellular; TM, transmembrane; IC, intracellular; black parts; human, white parts; bovine or cynomolgus.

The following human CD89 constructs were generated for epitope mapping and transiently expressed (1): membrane full-length human CD89 construct, which contained the full-length EC1 domain of human CD89 (SEQ 1), and therefore denoted as ‘human EC1-CD89’ (2). membrane chimeric human CD89/cynomolgus monkey CD89 construct (Gln22 – Lys46 from human EC1-CD89) (SEQ 13), and therefore denoted as ‘™Gln22 – Lys46 human EC1-CD89’ (3). membrane chimeric human CD89/cynomolgus monkey CD89 construct (Ile47 – Ile71 from human EC1-CD89) (SEQ 15), and therefore denoted as ‘™Ile47 – Ile71 human EC1-CD89’ (4). membrane chimeric human CD89/cynomolgus monkey CD89 construct (Gly72 – Gly96 from human EC-CD89) (SEQ 17), and therefore denoted as ‘™Gly72 – Gly96 human EC1-CD89’ (5). membrane chimeric human CD89/cynomolgus monkey CD89 construct (Arg97 – Gly121 from human EC1-CD89) (SEQ 19), and therefore denoted as ‘™Arg97 – Gly121 human EC1-CD89’ (6). membrane full-length cynomolgus monkey CD89 construct, which contained the full-length EC1 domain of human cynomolgus monkey CD89 (SEQ 11), and therefore denoted as ‘cynomolgus EC1-CD89’. cDNAs encoding for above-described ‘human EC1-CD89’, ‘™Gln22 – Lys46 human EC1-CD89’, ‘™Ile47 – Ile71 human EC1-CD89’, ‘™Gly72 – Gly96 human EC1-CD89’, and ‘cynomolgus EC1-CD89’ constructs were optimized for mammalian expression and synthesized by GENEART, Regensburg, Germany (SEQs 2, 14, 16, 18, 20, and 12, respectively). These cDNAs were subcloned in pcDNA3.1-derived expression plasmids as well. Detailed information about SEQs is shown in **Supplement Figure S2**.

Using the FreeStyle™ 293 Expression System (Invitrogen), FreeStyle™ HEK293F cells (Invitrogen) were transiently transfected with the different constructs. After 48-72 hours, flow cytometry analyses were performed to determine binding of mouse anti-human CD89 antibodies to chimeric human/bovine Fc receptors (domain mapping) and chimeric human CD89/cynomolgus monkey Fc receptors (epitope mapping) on transfected cells.

Transient transfected HEK293F cells were put at 10 x 10^6^ cells/mL in ice-chilled phosphate-buffered saline containing 0.1% BSA (Sigma)/0.05% NaN_3_ (PBS/BSA/NaN_3_) supplemented with 50 µg/mL human IgGs (blocking Fcγ receptors; Sigma) for 10 minutes at 4°C. Then, 10 μL/tube (i.e., 0.1 x 10^6^ cells) of these cells were incubated with or without 100 μL purified mouse anti-human CD89 antibody at 10 μg/mL (in PBS/BSA/NaN_3_) for 30 minutes at 4°C. In parallel, 100 μL purified mouse IgG1 isotype control (BD Biosciences) at 10 μg/mL (in PBS/BSA/NaN_3_) was used as a negative control, and 100 μL at 10 μg/mL (in PBS/BSA/NaN_3_) purified mouse anti-human CD89 antibody clone MIP8a (BioRad), clone A59 (BD Biosciences), and clone A3 (Santa Cruz Biotechnology) were used as positive controls. After extensive washing in PBS/BSA/NaN_3,_ cells were incubated with 1:200 diluted PE-conjugated goat anti-mouse IgG Fcγ-specific antibodies (Jackson ImmunoResearch) for 30 minutes at 4°C. After extensive washing in PBS/BSA/NaN_3_, cells were fixed in 2% formaldehyde in PBS/BSA/NaN_3_ for 30 minutes at 4°C. Binding of antibodies was measured with flow cytometry (BD FACSCalibur; BD Biosciences), and is expressed as signal-to-noise (S/N) ratios, i.e., dividing measured geometric fluorescence intensity of anti-CD89 antibody by measured geometric fluorescence intensity of mouse IgG1 isotype control.

### Immunofluorescence and analysis of tissue micro arrays

Cryosections of human TMAs (TSP-TMA-001, Tissue Solutions, Glasgow, UK) were kept at −80°C. Tissue sections (5 μm) were fixed with acetone for 10 minutes at RT, air dried, encircled with a hydrophobic pen, washed in PBS and blocked with PBS containing 2% BSA and 10% normal goat serum for 30 min at RT. Tissues were stained with mouse anti-hCD89 IgG1 clone 10E7, 20B4, 30C7 (Polpharma Biologics, Utrecht, The Netherlands) and mouse IgG1 isotype control (Biolegend, 400166). Subsequently, neutrophils were stained with Alexa fluor 488-conjugated mouse IgM anti-CD66b (NovusBio, NB100-77808AF488) and Alexa fluor 488-conjugated mouse IgM isotype control (Biolegend, 401617 AF488). Primary antibodies were diluted to 10,0 µg/ml and incubated overnight at 4°C. Alexa fluor 647-conjugated goat anti-mouse-IgG1 (Invitrogen, A-21236) was used as secondary antibody at 10,0 µg/ml and incubated for 1 hour at RT. Slides were washed, and nuclei were counterstained with 4’,6-Diamidino-2-Phenylindole, Dihydrochloride (DAPI, 1:5000) for 5 minutes at RT and slides were embedded with fluorescence mounting medium.

Stained sections were microscopic scanned with the Vectra^®^ Polaris™ (Akoya Biosciences) at a 20x magnification. Images were manually analyzed with Phenochart software (Akoya Biosciences). Isotype control staining’s of the mouse IgG1 and mouse IgM were compared with the hCD89 co-staining with CD66b to investigate whether neutrophils were stained with the hCD89 antibodies of Polpharma Biologics.

### *In vivo* linear IgA bullous disease mouse model

Transgenic mice expressing human CD89 and knock-in for human IgA were subcutaneously (sc) injected with 10 µl (7mg/ml) human-anti-mouse Collagen XVII IgA (auto)antibodies (Amsterdam UMC and Polpharma Biologics Utrecht) in the right ear and 10 µl PBS in the left ear as control. Injections were performed on day 0, 2, 4, 6, 8, 10 and 12. Mice were treated with 100 µl (1.5 mg/ml) of anti-human CD89 mouse antibody clone 10E7 (n=8, m/f) or a mouse IgG_1_ negative isotype control (Biolegend) (n=4, m/f). Antibodies were injected intraperitoneally at day 7 and 11. Mice were monitored daily for discomfort. At the end of the experiment, day 14, mice were sacrificed, ears were excised and subsequently snap-frozen to use for cryosectioning and immunofluorescence staining.

### Immunofluorescence and quantification of LABD ears

Mice ear cryosections (6 µm) were fixed in acetone for 10 min at RT and air-dried. Then, cryosections were incubated with 400x diluted Alexa Fluor 488-conjugated rat-anti-mouse Ly-6G (GR-1 staining, eBioscience) for 1 hour at RT. After washing in PBS, nuclei were stained using DAPI (Invitrogen) at 1 µg/ml for 5 minutes at RT and embedded with fluorescence mounting medium. Tile scanning to obtain an image of the whole ear was performed using the Vectra^®^ Polaris™ (Akoya Biosciences) microscope with the following settings: DAPI MSI 0.43ms, FITC 81.70ms and a 20 times magnification. GR-1 staining of cryosections was analyzed with ImageJ/Fiji software. The total area (µm^2^) of mice ears and the area of the specific GR-1 staining (µm^2^) was measured. Quantification was calculated as the GR-1 area (µm^2^) divided by the total area (µm^2^). The following formula was used to determine the GR-1/total area ratio:


$$\frac{\text{GR}-1\text{ Area }\left({\text{µm}}^{2}\right)}{\text{Total area }\left({\text{µm}}^{2}\right)/10,000\left({\text{µm}}^{2}\right)}$$

### Statistical analysis

Statistical analysis was performed using graph pad prism 9.1.0. In order to determine the differences in GR-1 area between treated and untreated mCOL17 ears, Mann-Whitney U test was performed. Result was considered significant if p< 0.05.

## Results

### Blocking anti-CD89 mAbs bind CD89 on neutrophils and inhibit IgA-induced neutrophil activation

**Figure 1:**
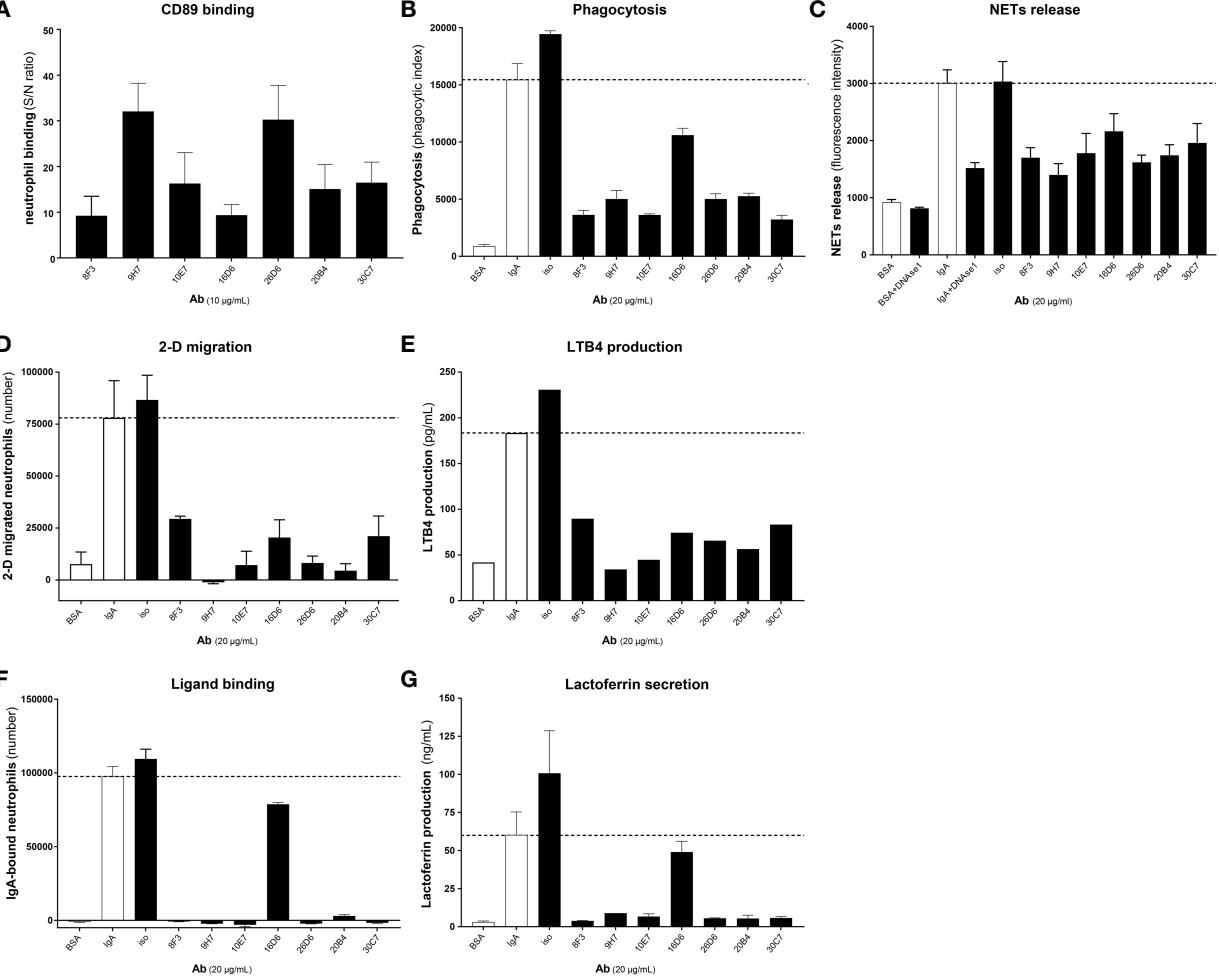
Developed anti-CD89 mAbs bind CD89 on neutrophils and inhibit IgA-induced neutrophil activation. **(A)** Binding of developed anti-CD89 mAbs to CD89 on neutrophils. **(B-F)** Phagocytosis of IgA-coated beads **(B)**, NETs release **(C)**, 2-D migration **(D)**, LTB4 production **(E)**, Ligand binding **(F)** and lactoferrin release (**G**; reflecting degranulation) in the absence or presence of anti-CD89 mAbs. BSA-coated beads served as negative control for aspecific phagocytosis of beads, and isotype mouse IgG1 was used as control for mAbs. In the NETs assay **(C)** DNase 1 was used as an additional control, as this will break down NETs. Representative results (means ± SD of replicates) from one representative donor are shown from at least three donors examined in each assay (i.e. Part labels **B, C, E–G**), except for FACS binding (i.e. Part label **A**), which is the mean ± SD of five donors and LTB4 production (i.e. Part label **D**), which was only performed on the supernatants (replicates pooled) of the representative donor. S/N-ratio; signal to noise ratio, CD89 Ab; antibody, NETs; neutrophil extracellular traps, LTB4; leukotriene B4.

First, the ability of the developed blocking anti-CD89 mAbs clones to bind CD89 on human neutrophils was analyzed. Isotype control mouse IgG1 was run in parallel as a negative control. Although some variation between the clones was seen, all tested anti-CD89 mAbs had the ability to bind CD89 (**Figure 1A**). Since the developed anti-CD89 mAbs effectively bound CD89 on primary immune cells, next we investigated whether IgA binding on neutrophils was prevented, and IgA-induced neutrophil activation subsequently inhibited.

Several functional assays were performed including phagocytosis, NETs-release, 2-D migration, IgA ligand binding and concomitant lactoferrin release. All anti-CD89 mAbs reduced IgA (coated to latex beads)-mediated phagocytosis and IgA induced NETs-release by CD89 expressing neutrophils, although clone 16D6 was relatively less effective (**Figures 1B, C**). Similarly, 2-D migration of neutrophils towards IgA coated sepharose beads was diminished in the presence of all blocking anti-CD89 mAbs (**Figure 1D**). Furthermore, a comparable pattern was observed for LTB4 release, a neutrophil chemoattractant, which was measured in the supernatant of 2-D migration assays (**Figure 1E**). Lastly, we determined the blocking capacity in a IgA ligand binding assay. The developed anti-CD89 mAbs blocked the binding of neutrophils to plate bound IgA (**Figure 1F**) and concomitant lactoferrin release, a measurement of degranulation, in the supernatant was reduced (**Figure 1G**). Again, clone 16D6 performed poorly in comparison with other mAbs, but collectively, these data show that the developed anti-CD89 mAbs have the capacity to bind CD89 on neutrophils and inhibit IgA-mediated functions.

### Anti-CD89 mAbs bind to different epitopes on the extracellular domain 1 of CD89

#### Domain mapping

**Figure 2:**
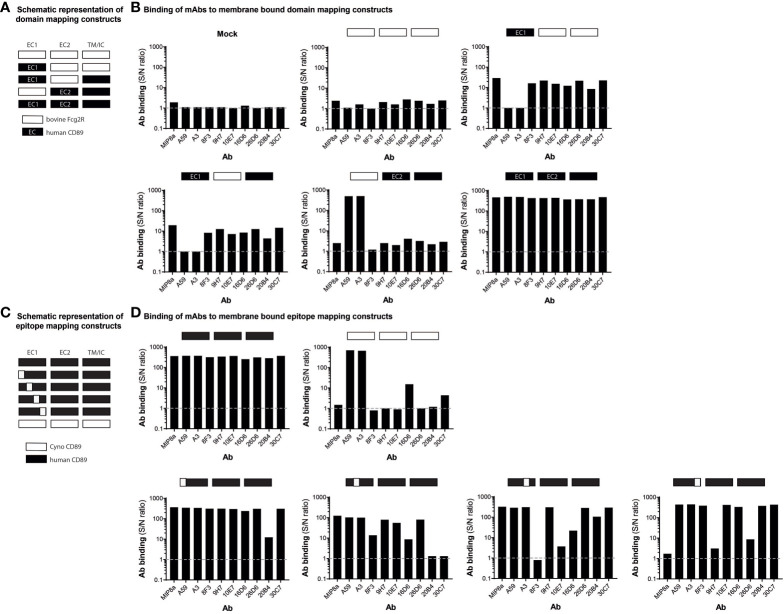
Anti-CD89 mAbs bind to different epitopes on extra cellular domain 1 of CD89 **(A)** Schematic representation of wild type human CD89, wild type bovine Fcγ2R, and their derived chimeric human/bovine FcR constructs (domain mapping). **(B)** Binding of mouse anti-human CD89 antibodies to membrane-bound human full-length CD89 (‘human EC1-EC2-CD89’ 

 

 

Structurally, human CD89 and bovine Fcγ2R are highly homologous and closely related to each other (34). To study to which domain our developed anti-CD89 mAbs bind, chimeric human/bovine receptors were designed by exchanging Ig-like EC1 and EC2 domains between these two receptor proteins (**Table 1**; **Figure 2A**). As expected, the developed anti-CD89 antibodies bound to full-length human CD89 (‘human EC1-EC2-CD89’) transfected cells but neither to mock-transfected cells nor to full-length bovine Fcγ2R transfected cells, which confirmed their specificity against human CD89 (**Figure 2B**). Moreover, anti-CD89 antibodies bound to both construct versions of ‘human EC1-CD89’ and not to ‘human EC2-CD89’ expressed on transfected 293F cells, which demonstrated that our anti-CD89 antibodies recognized epitopes within the EC1 domain of CD89. Commercially available mouse anti-human CD89 antibody clone MIP8a, which is known to recognize an epitope within the EC1 domain of human CD89 (35), bound to ‘human EC1-CD89’ and not to ‘human EC2-CD89’, whereas commercially available mouse anti-human CD89 antibody clone A59, which recognizes an epitope within the EC2 domain of human CD89 (36), bound to ‘human EC2-CD89’ and not to ‘human EC1-CD89’. Surprisingly, commercially available mouse anti-human CD89 antibody clone A3, which recognizes an epitope depending on parts from both EC1 and EC2 domains of human CD89 (36), bound to ‘human EC2-CD89’ but not to ‘human EC1-CD89’. This is in contrast to earlier findings in which it was demonstrated that clone A3 (partly) interacted with EC1 (36).

), to membrane-bound chimeric human EC1-CD89/bovine EC2-Fcγ2R (‘human EC1-CD89’ 

 
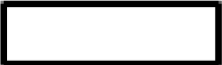
 
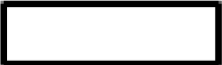
 and 

 
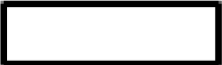
 

), to membrane-bound chimeric bovine EC1-Fcγ2R /human EC2-CD89 (‘human EC2-CD89’ 
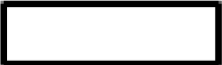
 

 

), and to membrane-bound bovine full-length Fcγ2R (’bovine Fcγ2R’ 
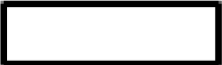
 
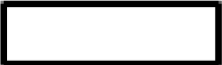
 
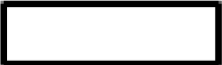
) on HEK293F cells. **(C)** Schematic representation of wild type human CD89, wild type cynomolgus monkey CD89, and their derived chimeric human/cynomolgus monkey CD89 constructs (epitope mapping). **(D)** Binding of mouse anti-human CD89 antibodies to membrane-bound human full-length CD89 (‘human EC1-CD89’; i.e., 

 

 

), to membrane-bound chimeric human/cynomolgus monkey CD89 (‘ΔGln22 – Lys46 human EC1-CD89’ , i.e., 
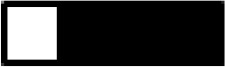
 

 

), to membrane-bound chimeric human/cynomolgus monkey CD89-II (‘ΔIle47 – Ile71 human EC1-CD89’, i.e., 
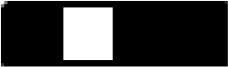
 

 

), to membrane-bound chimeric human/cynomolgus monkey CD89-III (‘ΔGly72 – Gly96 human EC1-CD89’, i.e., 
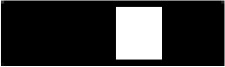
 

 

), to membrane-bound chimeric human/cynomolgus monkey CD89-IV (‘ΔArg97 – Gly121 human EC1-CD89’, i.e., 
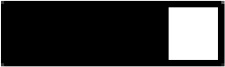
 

 

), and to membrane-bound chimeric cynomolgus monkey full-length CD89 (‘cynomolgus EC1-CD89’, i.e., 
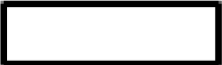
 
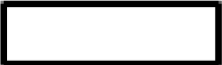
 
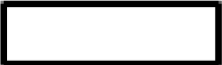
) on HEK293F cells. Dotted grey lines represent background (i.e., no binding of mouse anti-human CD89 antibodies). S/N; signal to noise, EC; extracellular, TM; transmembrane, I; intracellular.

#### Epitope mapping

Structurally, full-length human CD89 and full-length cynomolgus monkey CD89 are highly homologous and closely related to each other (37). Our generated anti- CD89 antibodies, which all recognized epitopes within the EC1 domain of human CD89 (see above), showed either no or weak cross-species reactivity with the EC1 domain of cynomolgus monkey CD89 in a pilot experiment (data not shown). Therefore, to study the epitope to which our anti-CD89 mAbs bind, chimeric human CD89/cynomolgus monkey CD89 receptors were designed by exchanging arbitrary parts (i.e., peptides of 25 amino acids in length) from the EC1 domain of human CD89 with reciprocal counterparts from the EC1 domain of cynomolgus monkey CD89 (**Table 1**; **Figure 2C**) to determine the regions/epitopes within the EC1 domain of human CD89, which are critical for the binding of our anti-CD89 antibodies. As anticipated, commercially available mouse anti-human CD89 antibody clone A59 and clone A3, which both recognize epitopes within the EC2 domain of human CD89 (see above), showed binding to examined chimeric human CD89/cynomolgus monkey CD89 constructs since the human EC2 domain was not changed in any of these chimeric constructs (**Figure 2D**). Moreover, both clones bound to full-length human CD89 and full-length cynomolgus monkey CD89, which illustrated that all constructs were expressed on the membrane surface of transiently transfected cells.

As shown in **Figure 2D**, the variation in binding profiles of our anti-CD89 antibodies against chimeric human/cynomolgus monkey CD89 constructs support that they recognized different epitopes within the EC1 domain of human CD89. For example, the anti-CD89 antibody clone 20B4 recognized an epitope located at the distal N-terminal region of the EC1 domain, while clones 9H7 and 26D6 recognized an epitope at the opposite site of the EC1 domain (i.e., adjacent to the EC2 domain), as did commercially available mouse anti-human CD89 antibody clone MIP8a. However, no competition experiments were performed to actually rule out that the antibodies 9H7, 26D6 and MIP8a, which lost binding after substitution of the same 25-amino acids peptide, were not binding to the same (or largely overlapping) epitopes. Finally, anti-CD89 mAbs clones 8F3, 10E7, 16D6, and 30C7 recognized epitopes in the ‘centre’ of the EC1 domain.

### Anti-CD89 antibody clone 10E7 does not cross-react with unrelated antigens in human tissues

**Figure 3:**
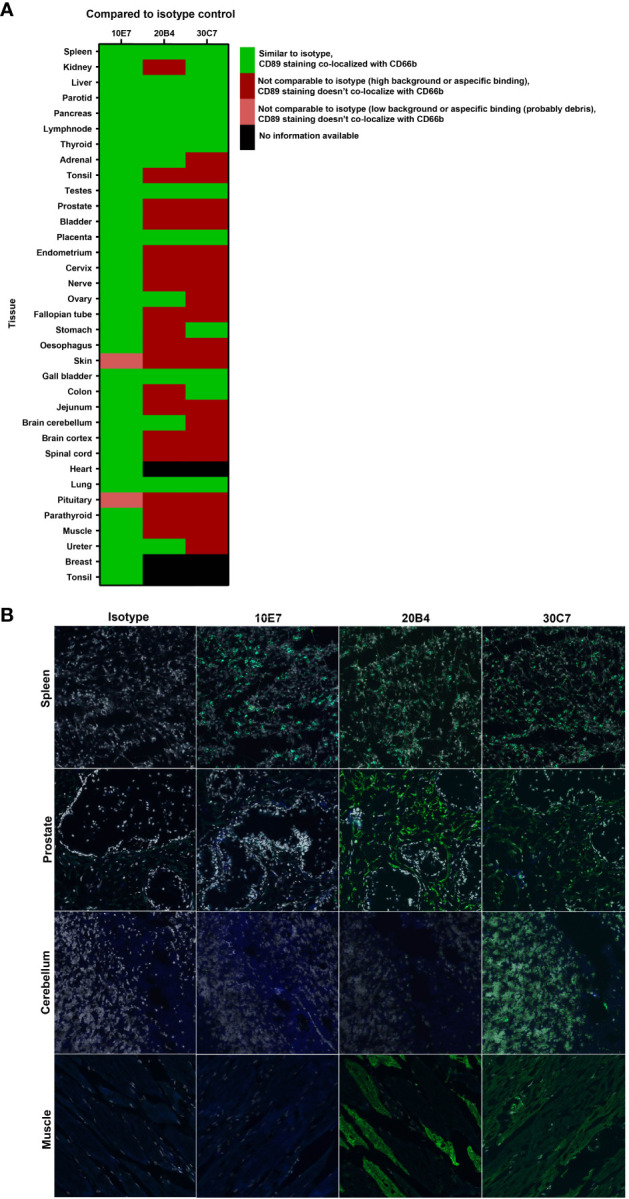
No cross-reactivity with anti-CD89 mAb clone 10E7 in normal human tissues **(A)** Heatmap of tissue micro array stainings that were performed and analyzed for specific binding to CD89 on neutrophils (CD66+) to check for cross-reactivity of lead anti-CD89 clones 10E7, 20B4 and 30C7. Staining was compared to isotype mouse IgG1 negative control. **(B)** All three examined clones bound CD89 expressed on CD66b+ neutrophils observed in lymphoid tissues (e.g. spleen, upper panels). However, unexpected cross-reactivity on CD66b-cells was found in e.g. prostate (second panels), cerebellum (third panels) and skeletal muscle (lower panels) after staining with clone 20B4 and/or 30C7. Dapi (grey; nucleus), CD66b (blue), CD89 (green), CD66b+CD89 (light green).

Based on the functional and epitope mapping studies, clones 10E7, 20B4 and 30C7 were selected for further evaluation, as these clones showed the best capacity to inhibit IgA-induced neutrophil activation and bound to a different epitope compared to MIP8a. Potential cross-reactivity of our lead anti-CD89 mAbs clone 10E7, 20B4 and 30C3 was determined on cryosections of normal human tissues. As expected, all clones bound to neutrophils in lymphoid tissues, which was exemplified by their double staining on CD66B^+^ cells (e.g. in spleen, **Figure 3B** and in tonsil tissue, see **Supplement Figure S3**). However, we observed unexpected cross-reactivity of 20B4 to e.g. prostate and skeletal muscle tissues, whereas 30C7 cross-reacted to e.g. prostate, cerebellum and skeletal muscle tissues (**Figures 3A, B**). Since this may lead to serious side effects, we concluded that it was unsafe to further develop clone 20B4 and 30C7. Some aspecific background staining of 10E7 was observed in the epidermis, but this was also seen for the isotype control. Similarly, some aspecific staining of both 10E7 and the isotype control was observed in the pituitary, which may have been debris as no co-localization with nuclei was present (see **Supplement Figure S4**). Thus clone 10E7 binding was restricted to CD66B^+^ neutrophils and some tissue macrophages. Therefore, clone 10E7 became our lead mAb for preclinical animal testing.

### Anti-CD89 mAb 10E7 blocks neutrophil influx in a mouse model for linear IgA bullous disease

**Figure 4:**
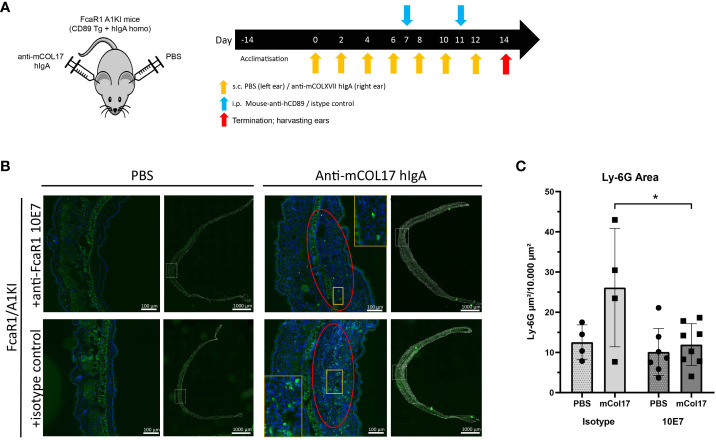
Anti-CD89 mAb 10E7 reduces neutrophil influx in a mouse model for linear IgA bullous disease. **(A)** Injection schedule of mice; Human IgA x human CD89 transgenic mice were injected 7 times with anti-mouse COLXVII human IgA in the right and PBS in the left ears. Treatment with anti-CD89 mAb clone 10E7 or the isotype mouse IgG1 control was given at day 7 and 11. **(B)** Neutrophil influx in PBS-injected ears (left panels) or human IgA anti-mouse COLXVII- injected ears (right panels) of human IgA x human CD89 transgenic mice that had been treated with anti-CD89 mAb 10E7 (upper panels) or isotype control (lower panels). Dapi (blue (in magnification) or grey (for overview ear)), GR-1 (green). **(C)** Quantification of neutrophil influx in ears. Mean ± SD are shown. Black circles and black squares represent number of neutrophils in PBS injected or anti-mouse collagen XVII human IgA injected ears of individual mice respectively. Mann-Whitney U tests,*; P<0.05, NS; not significant, mAb; monoclonal antibody, LABD; linear IgA bullous disease, COL; collagen, PBS; phosphate buffered saline, SD; standard deviation.

To investigate whether anti-CD89 mAb clone 10E7 blocks IgA induced neutrophil influx and tissue damage *in vivo*, we used a preclinical mice model for LABD (18) using in-house bred human IgA knock in x human CD89 transgenic mice (32, 33) (**Figure 4A**). We make use of this mice model since wild type mice do not express (an equivalent receptor for human) CD89 (FcαR1) on their immune cells, precluding functional studies of the role of CD89 in wild type mice. Previous characterization of CD89 transgenic mice showed that human CD89 is expressed on neutrophils, on a subpopulation of monocytes and on macrophages after activation (32). To further mimic the human situation, mice were crossbred with human IgA knock-in mice (32, 33). Injection of human IgA anti-mouse COLXVII (the autoantigen in LABD) in the ears of human IgA knock in x human CD89 transgenic mice resulted in neutrophil influx in the skin (for injection schedule, see **Figure 4A**), which was significantly reduced after treatment with anti-CD89 mAb clone 10E7 on day 7 and 11 (**Figures 4B, C**).

These results demonstrated that anti-CD89 mAb clone 10E7 had the ability to reduce experimentally induced human IgA-mediated neutrophil influx in human IgA x human CD89 transgenic mice.

## Discussion

IgA is a potent stimulus to activate neutrophils after crosslinking of their surface CD89 (6, 7). As such, we anticipate that excessive activation of neutrophils by IgA complexes or IgA autoantibodies contributes to chronic inflammation and tissue damage, and may therefore be involved in disease pathology including LABD, DH and RA.

Especially, neutrophil activation in LABD is a direct result of CD89 triggering by IgA autoantibodies directed against collagen type XVII, which leads to skin damage, blister formation, and itchy skins (16, 18). The current first line treatment option for LABD is Dapsone, a leprostatic agent, although the exact therapeutic mechanism is unknown (38, 39). Patients who do not tolerate Dapsone, can be treated with sulfonamides (40), colchicine (41) or glucocorticoids like prednisone (42). DH is another skin blistering disease. Patients have IgA autoantibodies against tissue transglutaminase, which cross-react with epidermal transglutaminase, resulting in IgA deposits and infiltration of neutrophils in the dermal papillae (19). The standard treatment for DH is Dapsone as well. For most patients (LABD and DH), long term therapy is needed, but serious side effects can occur after long-term usage of the current medicines. Therefore, more specific treatment options are needed.

In RA, IgA autoantibodies are present and neutrophils are abundantly present in synovial fluid of affected joints (29, 30). High IgA ACPA and IgA RF titers correlate with a more severe disease outcome and can be used as a predictive biomarker for disease progression (24–26). Current treatment of RA patients is based on suppressing the immune system and starts normally with disease modifying anti-rheumatic drugs (DMARDS), like methotrexate. If patients fail to respond, biologicals such as Rituximab and anti-TNF can be included as treatment options. However, about one third of all patients do not respond to e.g. anti-TNF treatment. Also in this case, new treatment opportunities are needed to overcome this problem. Since studies showed an association between high levels of RF IgA and poor clinical response to TNF blockers as well as an association of IgA autoantibodies with a more severe disease course (24, 26, 43), interfering with the CD89-hIgA pathway might be a treatment option.

We now demonstrate that our newly developed anti-human CD89 mAbs effectively inhibited IgA-induced human neutrophil activation in various functional *in vitro* assays. Although they bound to different epitopes on EC1 of CD89, all blocking antibodies were able to diminish IgA induced phagocytosis, migration and NETs release. Furthermore, our lead anti-CD89 mAb clone 10E7 did not show unexpected cross-reactivity with other antigens in human tissues and resolved existing inflammation in a preclinical mouse model for LABD. More research in preclinical RA and DH mouse models is necessary to determine the therapeutic potential of our lead anti-CD89 mAb clone 10E7 for these diseases.

A limitation of the study is that all experiments were performed using neutrophils isolated from fresh healthy donor blood. In (systemic) auto-immune diseases, neutrophils might be primed due to the pro-inflammatory circumstances. Further research is necessary to investigate whether disease specific altered functions are induced by IgA stimulation and whether blocking CD89 can still inhibit IgA mediated effects in diseases. Another limitation of the study is the TMA staining, in which all tissues were stained using the same protocol. For some tissues a slightly adapted staining protocol might have been more optimal to prevent background staining or auto-fluorescence.

In summary, these data demonstrate a promising novel therapy for patients with LABD and probably for patients with other autoimmune diseases (e.g. DH, RA) in which IgA autoantibodies play a role as well. Moreover, in these diseases IgA autoantibodies may serve as a biomarker for disease severity. Since IgA autoantibodies can routinely be determined in these patients as predictive biomarker, high levels of IgA autoantibodies can be used as companion diagnostic to stratify patients for treatment with antagonist anti-CD89 mAbs, enabling a personalized medicine approach in these diseases. Treatment with anti-CD89 mAbs will likely have to be repeated, since IgA autoantibodies will remain present. However, frequency and dose are yet unknown and need to be further elucidated in (pre)clinical (human) studies. The frequency and dose might depend on various factors like age, weight and the amount of soluble CD89 in the circulation. Possible side effects after treatment might be a higher risk for infections in the airways and gastrointestinal track. Although most IgA deficient people do not experience increased infections, probably due to the development of a back-up system, we don’t know whether this will be the case in patients in which the IgA-CD89 pathway is blocked later in life. Further research and follow up is needed to study this.

To conclude, this study indicates that our newly developed anti-CD89 mAbs inhibited IgA-induced neutrophil activation and, most importantly, our anti-CD89 mAb clone 10E7 reduced anti-autoantigen IgA-induced neutrophil influx in an *in vivo* LABD model.

## Data availability statement

Inquiries for the availability of the raw data supporting the conclusions of this article can be directed to the corresponding author.

## Ethics statement

All human FF and granulosa cell specimens were obtained from the CReATe Biobank, CReATe Fertility Centre with written informed consent. The CReATe Biobank (banking protocols approved by Veritas IRB (Approval#16518), collects biological materials from consenting patients, according to the best practice-based standards of biobanking. All samples from the Biobank were approved for use in this study by the Veritas IRB (Approval#16487) and The Ottawa Hospital REB (Protocol #20170453-01H)]. The patients/participants provided their written informed consent to participate in this study. All animal procedures were carried out in accordance with the Guidelines for the Care and Use of Laboratory Animals, Canadian Council on Animal Care, and were approved by the University of Ottawa Animal Care Committee.

## Author contributions

MvD, EA, LB, PS and MvE contributed to conception and design of the study. MvD, EA, RM, and NdJ performed the experiments and analisys. MvD wrote the first draft of the manuscript. MvD, RM, LB and PS wrote sections of the manuscript. All authors contributed to manuscript revision, read, and approved the submitted version.
