# Supplementary material for: Antagonizing FcαR1 (CD89) as treatment in IgA-mediated chronic inflammation and autoimmunity
Source: Front Immunol. 2023 Apr 4;14:1118539. doi: 10.3389/fimmu.2023.1118539 (PMC10111428; doi:10.3389/fimmu.2023.1118539)
Supplement: Supplementary file 1 [file Presentation_1.pdf]

Supplemental material

S1. Description of chimeric human CD89/bovine Fcγ2R sequences.

SEQ 1:

Amino acid sequence of full-length human CD89 (Swiss-Prot no. P24071.1; aa 287)

MDPKQTTLLCLVLCLGQRIQAQEGDFPMPFISAKSSPVIPLDGSVKIQCQAIREAYLTQLMIIKNSTYREIGRRLKFWNETDPEFVIDHMDANKAGRY
QCQYRIGHYRFRYSDTLELVVTGLYGKPFLSADRGLVLMPGENISLTCSSAHIPFDRFSLAKEGELSLPQHQSGEHPANFSLGPVDLNVSGIYRCYGW
YNRSPYLWSFPSNALELVVTDSIHQDYTTQNLIRMAVAGLVLVALLAILVENWHSHTALNKEASADVAEPSWSQQMCQPGLTFARTPSVCK

Signal peptide (aa sequence 1-21), extracellular domain (aa sequence 22-227), comprising of an Ig-like EC1 domain (aa sequence 22-121), of a short hinge region (aa sequence 122-125), of an Ig-like EC2 domain (aa sequence 126-220), and of a membrane proximal 'linker' region (aa sequence 221 -227), followed by transmembrane domain (aa sequence 228-246), and short cytoplasmic tail (aa sequence 247-287) according to Ding et al. J Biol Chem 2003; 278:27966-27970.

SEQ 2:

cDNA sequence coding for full-length human CD89 protein (optimized for mammalian expression)

atggaccccaagcagaccaccctgctgtgcctggtgctgtgtctgggccagagaatccaggcccaggaaggcgacttccccatgcccttcatcagcgccaagagcagccccgtgatcccc
ctggatggcagcgtgaagatccagtgccaggccatcagagaggcctacctgacccagctgatgatcattaagaacagcacctaccgcgagatcggcagacggctgaagttctggaacg
agacagaccccgagttcgtgatcgaccacatggacgccaacaaggccggcagataccagtgtcagtaccggatcggccactaccggttccggtacagcgacaccctggaactggtcgtg
accggcctgtacggcaagcctttcctgagcgccgatcggggactggtgctgatgcccggcgagaacatcagcctgacctgtagcagcgcccacatccccttcgacagattcagcctggcc
aaagagggcgagctgagcctgcctcagcatcagtctggcgagcaccccgccaactttagcctgggccctgtggacctgaacgtgtccggcatctaccggtgctacggctggtacaaccgg
tccccctacctgtggtccttccccagcaacgctctggaactggtcgtgacagacagcatccaccaggactacaccacccagaacctgatccggatggccgtggctgggctggtgctggtg
gctctgctggccattctggtggaaaactggcacagccacaccgccctgaacaaagaggccagcgccgatgtggccgagccttcttggagccagcagatgtgtcagcccggcctgaccttc
gccagaaccccttctgtgtgcaag

SEQ 3:

Amino acid sequence of chimeric FcR comprising EC1 from human CD89 and EC2 from bovine Fcγ2R (bovine TM & bovine IC)

MDPKQTTLLCLVLCLGQRIQAQEGDFPMPFISAKSSPVIPLDGSVKIQCQAIREAYLTQLMIIKNSTYREIGRRLKFWNETDPEFVIDHMDANKAGRY
QCQYRIGHYRFRYSDTLELVVTGEEPAGRLRDRPSLSVRPSPSVAPGENVTLLCQSGNRTDTFLLSKEGAAHRPLRLRSQDQDGWYQAEFSLSPVTS
AHGGTYRCYRSLSTNPYLLSQPSEPLALLVADYTMQNLIRMGLAASVLLLLGILLCQARHDHGGAREAARS

Signal peptide from human CD89 (aa sequence 1-21), chimeric human/bovine FcR extracellular domain (aa sequence 22-232), consisting of the EC1 domain from human CD89 (aa sequence 22-121) and of the EC2 domain from bovine Fcγ2R (aa sequence 122-232), transmembrane domain and short cytoplasmic tail from bovine Fcγ2R (aa sequence 233-266).

SEQ 4:

Amino acid sequence of chimeric FcR comprising EC1 from human CD89 and EC2 from bovine Fcγ2R (human TM & human IC)

MDPKQTTLLCLVLCLGQRIQAQEGDFPMPFISAKSSPVIPLDGSVKIQCQAIREAYLTQLMIIKNSTYREIGRRLKFWNETDPEFVIDHMDANKAGRY
QCQYRIGHYRFRYSDTLELVVTGEEPAGRLRDRPSLSVRPSPSVAPGENVTLLCQSGNRTDTFLLSKEGAAHRPLRLRSQDQDGWYQAEFSLSPVTS
AHGGTYRCYRSLSTNPYLLSQPSEPLALLVADYTMQNLIRMAVAGLVLVALLAILVENWHSHTALNKEASADVAEPSWSQQMCQPGLTFARTPSV
CK

Signal peptide from human CD89 (aa sequence 1-21), chimeric human/bovine FcR extracellular domain (aa sequence 22-232), consisting of the EC1 domain from human CD89 (aa sequence 22-121) and of the EC2 domain from bovine Fcγ2R (aa sequence 122-232), transmembrane domain from human CD89 (aa sequence 233-251), and short cytoplasmic tail from human CD89 (aa sequence 252-292).

SEQ 5:

cDNA sequence coding for chimeric FcR comprising EC1 from human CD89 and EC2 from bovine Fcγ2R (bovine TM & bovine IC; optimized for mammalian expression)

atggaccccaagcagaccacactgctgtgcctggtgctgtgtctcggccagagaatccaagctcaagagggcgacttccccatgcctttcatcagcgccaagagcagccctgtgatccct
ctggatggcagcgtgaagatccagtgccaggccatcagagaggcctacctgacacagctgatgatcattaagaacagcacctaccgcgagatcggcagacggctgaagttctggaacg
agacagaccccgagttcgtgatcgaccacatggacgccaacaaggccggcagataccagtgtcagtaccggatcggccactaccggttcagatacagcgacaccctggaactggtggt
caccggcgaagaacctgctggcagactgagagatagacccagcctgtctgtgcggccttctccttctgttgcccctggcgagaatgtgaccctgctctgtcagagcggcaaccggaccga
taccttcctgctgtctaaagaaggcgccgctcacagacccctgagactgagatcacaggaccaggacggatggtatcaggccgagttctctctgagcccagtgacatctgctcacggcgg
cacctacagatgctacagaagcctgagcacaaacccctatctgctgagccagcctagcgagcctctggctctgctggtggccgattacaccatgcagaacctgatcagaatgggcctcgc
cgcctctgttctgctgctgctgggaatcctgctgtgtcaagccagacacgatcacggcggagccagagaagctgccagatct

SEQ 6:

cDNA sequence coding for chimeric FcR comprising EC1 from human CD89 and EC2 from bovine Fcγ2R (human TM & human IC; optimized for mammalian expression)

atggaccccaagcagaccacactgctgtgcctggtgctgtgtctcggccagagaatccaagctcaagagggcgacttccccatgcctttcatcagcgccaagagcagccctgtgatccct
ctggatggcagcgtgaagatccagtgccaggccatcagagaggcctacctgacacagctgatgatcattaagaacagcacctaccgcgagatcggcagacggctgaagttctggaacg
agacagaccccgagttcgtgatcgaccacatggacgccaacaaggccggcagataccagtgtcagtaccggatcggccactaccggttcagatacagcgacaccctggaactggtggt
caccggcgaagaacctgctggcagactgagagatagacccagcctgtctgtgcggccttctccttctgttgcccctggcgagaatgtgaccctgctctgtcagagcggcaaccggaccga
taccttcctgctgtctaaagaaggcgccgctcacagacccctgagactgagatcacaggaccaggacggatggtatcaggccgagttctctctgagcccagtgacatctgctcacggcgg
cacctacagatgctacagaagcctgagcacaaacccctatctgctgagccagcctagcgagcctctggctctgctggtggccgattacaccatgcagaacctgatcagaatggccgtggc
cggactggtgctggttgcactgctggctatcctggtggaaaactggcacagccacacagccctgaacaaagaggcttctgccgacgtcgccgagccttcttggagtcagcagatgtgtca
gcccggcctgaccttcgccagaacacctagcgtgtgcaag

SEQ 7:

Amino acid sequence of chimeric FcR comprising EC1 from bovine Fcγ2R and EC2 from human CD89 (human TM & human IC)

MAPTLPALLCLGLSVGLRTQVQAGTFPKPIIWAEPSSVVPLGSSVTILCQGPPNTKSFSLNKEGDSTPWNIHPSLEPWDKANFFISNVREQQAGRYH
CSHFIGVNWSEPSEPLDLLVAGLYGKPFLSADRGLVLMPGENISLTCSSAHIPFDRFSLAKEGELSLPQHQSGEHPANFSLGPVDLNVSGIYRCYGWY
NRSPYLWSFPSNALELVVTDSIHQDYTTQNLIRMAVAGLVLVALLAILVENWHSHTALNKEASADVAEPSWSQQMCQPGLTFARTPSVCK

Signal peptide from bovine Fcγ2R (aa sequence 1-23), chimeric bovine/human FcR extracellular domain (aa sequence 24-225), consisting of the EC1 domain from bovine Fcγ2R (aa sequence 24-119), of the short hinge region from human CD89 (aa sequence aa 120-123), of the EC2 domain from human CD89 (aa sequence 124 -218), and of the membrane proximal ‘linker’ region from human CD89 (aa sequence 219 -225), followed transmembrane domain from human CD89 (aa sequence 226-244), and short cytoplasmic tail from human CD89 (aa sequence 245-285) according to Ding et al. J Biol Chem 2003; 278:27966-27970.

SEQ 8:

cDNA sequence coding for chimeric FcR chimeric FcR comprising EC1 of bovine Fcγ2R and EC2 of human CD89 (optimized for mammalian expression)

atggcccctacactgcctgctctgctgtgtctgggactgtctgtgggcctgagaacacaggtgcaggccggcacattccccaagcctatcatttgggccgagcctagctctgtggtgcctct
gggaagcagcgtgaccatcctgtgtcagggccctccaaacaccaagagcttcagcctgaacaaagagggcgacagcaccccttggaacattcaccctagcctggaaccttgggacaaa
gccaacttcttcatcagcaacgtgcgcgagcagcaggccggaagataccactgctctcacttcatcggagtgaattggagcgagcccagcgagcctctggatctgcttgtggctggcctgt
acggcaagccttttctgtctgccgatagaggcctggtgctgatgcccggcgagaatatcagcctgacctgtagcagcgctcacatccccttcgacagattctccctggccaaagaaggcga
gctgagcctgcctcagcatcagtctggcgaacaccccgccaacttttctctgggccctgtggacctgaacgtgtccggcatctacagatgctacggctggtacaatcggagcccctacctgt
ggtctttccccagcaatgccctggaactggtggtcaccgatagcatccaccaggactacaccacacagaacctgatcagaatggccgtggccggactggtgctggttgcactgctggcta
ttctggtggaaaactggcacagccacaccgctctcaacaaagaagcctctgccgacgtcgccgagccttcttggagtcagcagatgtgtcagcccggcctgaccttcgccagaacaccta
gcgtgtgcaag

SEQ 9:

Amino acid sequence of full-length bovine Fcγ2R (Swiss-Prot no. Q28109; aa 264)

MAPTLPALLCLGLSVGLRTQVQAGTFPKPIIWAEPSSVVPLGSSVTILCQGPPNTKSFSLNKEGDSTPWNIHPSLEPWDKANFFISNVREQQAGRYH
CSHFIGVNWSEPSEPLDLLVAGEEPAGRLRDRPSLSVRPSPSVAPGENVTLLCQSGNRTDTFLLSKEGAAHRPLRLRSQDQDGWYQAEFSLSPVTSA
HGGTYRCYRSLSTNPYLLSQPSEPLALLVADYTMQNLIRMGLAASVLLLLGILLCQARHDHGGAREAARS

Signal peptide (aa sequence 1-23), extracellular domain (aa sequence 24-230), comprising of an Ig-like EC1 domain (aa sequence 24-119) and of an Ig-like EC2 domain (aa sequence 120-230), followed by transmembrane domain and short cytoplasmic tail (aa sequence 231-264).

SEQ 10:

cDNA sequence coding for full-length bovine Fcγ2R protein (optimized for mammalian expression)

atggcccctacactgcctgctctgctgtgtctgggactgtctgtgggcctgagaacacaggtgcaggccggcacattccccaagcctatcatttgggccgagcctagctctgtggtgcctct
gggaagcagcgtgaccatcctgtgtcagggccctccaaacaccaagagcttcagcctgaacaaagagggcgacagcaccccttggaacattcaccctagcctggaaccttgggacaaa
gccaacttcttcatcagcaacgtgcgcgagcagcaggccggaagataccactgctctcacttcatcggagtgaattggagcgagcccagcgagcctctggatctgcttgttgctggcgaa
gaaccagccggcagactgagagatagaccctctctgagtgtgcggccctctccttctgttgcccctggcgaaaatgtgaccctgctgtgccagagcggcaacaggaccgataccttcctgc
tgagcaaagaaggcgccgctcacagaccccctgagactgagatcacaggaccaggacggatggtatcaggccgagttcagcctgtctcctgtgacatctgctcacggcggcacctacaga
tgctacagaagcctgagcacaaacccctacctgctgtcccagccttctgagcctttggctctgctggtggccgactacaccatgcagaacctgatcagaatgggcctcgccgcctctgttct
gctgctgctgggaatcctgctctgtcaggccagacacgatcatggcggagccagagaagccgccagatct

S2. Description of chimeric human CD89/cynomolgus monkey CD89 sequences.

SEQ 11:

Amino acid sequence of full-length cynomolgus monkey CD89 (NCBI Reference Sequence: XP_005590398.1; aa 287)

MDPKETTLLCLVLCLGQRIQAQEGNFSTPFISTRSSPVVPWGGSVRIQCQAIPDAYLIWLMMLKNSTYEKRDEKLGFWNDTTPEFVIDHMDANKA
GRYRCRYRIGLSRFRYSDTLELVVTGLYGKPSLSVDRGPVLMPGENISVTCSSAHIPFDRFSLAKEGELSLPQHQSGEHPANFSLGPVDLNVSGSYRCY
GWYNRSPYLWSFPSNALELVVTDSINRDYTTQNLIRMAMAGLVLVALLAILVENWHSHKALNKEASADVAEPSWSHQMCQPGWTFARTPSVCK

SEQ 12:

cDNA sequence coding for full-length cynomolgus monkey CD89 (optimized for mammalian expression)

atggaccccaaagaaaccaccctgctgtgcctggtgctgtgtctgggccagagaatccaggcccaggaaggcaacttcagcaccccсttcatcagcaccagatccagccccgtggtgcct
tggggaggctctgtgcggattcagtgccaggccatccccgacgcctacctgatctggctgatgatgctgaagaacagcacctacgagaagcgggacgagaagctgggcttctggaacga
caccacccccgagttcgtgatcgaccacatggacgccaacaaggccggcagataccggtgccggtacagaatcggcctgagccggttcagatacagcgacaccctggaactggtcgtg
accggcctgtacggcaagcctagcctgtccgtggatagaggccccgtgctgatgcccggcgagaacatcagcgtgacctgtagcagcgcccacatccccttcgacagattcagcctggcc
aaagagggcgagctgagcctgcctcagcatcagtctggcgagcaccccgccaactttagcctgggccctgtggacctgaacgtgtccggcagctacagatgctacggctggtacaaccg
gtcccсctacctgtggtccttccccagcaacgctctggaactggtcgtgacagacagcatcaaccgggactacaccacccagaacctgatccggatggctatggccggactggtgctggt
ggccctgctggccatcctggtggaaaactggcacagccacaaggccctgaacaaagaggccagcgccgatgtggccgagccttcttggagccaccagatgtgtcagcccggctggacct
tcgccagaaccccttctgtgtgcaag

SEQ 13:

Amino acid sequence of chimeric CD89 by exchanging EC1 part from human CD89 (Gln22 – Lys46) with EC1 part from cynomolgus monkey CD89 (**Gln22 – Arg46**)

MDPKQTTLLCLVLCLGQRIQAQEGNFSTPFISTRSSPVVPWGGSVRIQCQAIREAYLTQLMIIKNSTYREIGRRLKFWNETDPEFVIDHMDANKAGR
YQCQYRIGHYRFRYSDTLELVVTGLYGKPFLSADRGLVLMPGENISLTCSSAHIPFDRFSLAKEGELSLPQHQSGEHPANFSLGPVDLNVSGIYRCYG
WYNRSPYLWSFPSNALELVVTDSIHQDYTTQNLIRMAVAGLVLVALLAILVENWHSHTALNKEASADVAEPSWSQQMCQPGLTFARTPSVCK

Signal peptide from human CD89 (aa sequence 1-21), chimeric human/cynomolgus monkey CD89 extracellular domain (aa sequence 22-227), comprising of an Ig-like EC1 domain part from cynomolgus monkey CD89 (aa sequence 22-46; NCBI Reference Sequence: XP_005590398.1) and an Ig-like EC1 domain part from human CD89 (aa sequence 47-121; Swiss-Prot no. P24071.1), of a short hinge region from human CD89 (aa sequence 122-125), of an Ig-like EC2 domain from human CD89 (aa sequence 126-220), and of a membrane proximal 'linker' region from human CD89 (aa sequence 221 -227), followed by transmembrane domain from human CD89 (aa sequence 228-246), and short cytoplasmic tail from human CD89 (aa sequence 247-287) according to Ding et al. J Biol Chem 2003; 278:27966-27970.

SEQ 14:

cDNA sequence coding for chimeric CD89 by exchanging EC1 part from human CD89 (Gln22 – Lys46) with EC1 part from cynomolgus monkey CD89 (**Gln22 – Arg46**) (optimized for mammalian expression)

atggaccccaagcagaccacactgctgtgcctggtgctgtgtctcggccagagaatccaggctcaagagggcaacttcagcacccctttcatcagcaccagatctagccccgtggtgcctt
ggggcggctctgttagaatccagtgccaggccatcagagaggcctacctgacacagctgatgatcattaagaacagcacctaccgcgagatcggcagacggctgaagttctggaacga
gacagaccccgagttcgtgatcgaccacatggacgccaacaaggccggcagataccagtgtcagtaccggatcggccactaccggttcagatacagcgacaccctggaactggtggtc
accggcctgtacggcaagcctttttctgagcgccgatagaggcctggtcctgatgcctggcgagaacatcagcctgacctgtagcagcgctcacatccccttcgacagattcagcctggcca
aagagggcgagctgtctctgcctcagcatcagtctggcgagcaccccgccaatttttctctgggccctgtggacctgaacgtgtccggcatctacagatgctacggctggtacaatcggag
cccctacctgtggtctttccccagcaatgccctcgaactggtcgtgaccgatagcatccaccaggactacaccacacagaacctgatcagaatggccgtggccggactggtgctggttgct
ctgctggctattctggtggaaaactggcacagccacacagccctgaacaaagaggcttctgccgacgtcgccgagccttcttggagtcagcagatgtgtcagcccggcctgaccttcgcc
agaacacctagcgtgtgcaag

SEQ 15:

Amino acid sequence of chimeric CD89 by exchanging EC1 part from human CD89 (Ile47 – Ile71) with EC1 part from cynomolgus monkey CD89 (**Ile47 – Arg71**)

MDPKQTTLLCLVLCLGQRIQAQEGDFPMPFISAKSSPVIPLDGSVKIQCQAIPDAYLIWLMMLKNSTYEKRGRRLKFWNETDPEFVIDHMDANKAG
RYQCQYRIGHYRFRYSDTLELVVTGLYGKPFLSADRGLVLMPGENISLTCSSAHIPFDRFSLAKEGELSLPQHQSGEHPANFSLGPVDLNVSGIYRCYG
WYNRSPYLWSFPSNALELVVTDSIHQDYTTQNLIRMAVAGLVLVALLAILVENWHSHTALNKEASADVAEPSWSQQMCQPGLTFARTPSVCK

Signal peptide from human CD89 (aa sequence 1-21), chimeric human/cynomolgus monkey CD89 extracellular domain (aa sequence 22-227), comprising of an Ig-like EC1 domain part from human CD89 (aa sequence 22-46; Swiss-Prot no. P24071.1), an Ig-like EC1 domain part from cynomolgus monkey CD89 (aa sequence 47-71; NCBI Reference Sequence: XP_005590398.1) and an Ig-like EC1 domain part from human CD89 (aa sequence 48-121; Swiss-Prot no. P24071.1), of a short hinge region from human CD89 (aa sequence 122-125), of an Ig-like EC2 domain from human CD89 (aa sequence 126-220), and of a membrane proximal 'linker' region from human CD89 (aa sequence 221 -227), followed by transmembrane domain from human CD89 (aa sequence 228-246), and short cytoplasmic tail from human CD89 (aa sequence 247-287) according to Ding et al. J Biol Chem 2003; 278:27966-27970.

SEQ 16:

cDNA sequence coding for chimeric CD89 by exchanging EC1 part from human CD89 (Ile47 – Ile71) with EC1 part from cynomolgus monkey CD89 (**Ile47 – Arg71**) (optimized for mammalian expression)

atggaccccaagcagaccacactgctgtgcctggtgctgtgtctcggccagagaatccaagctcaagagggcgacttccccatgcctttcatcagcgccaagagcagccctgtgatccct
ctggatggcagcgtgaagatccagtgccaggccattcctgacgcctacctgatctggctgatgatgctgaagaacagcacctacgagaagagaggcagacggctgaagttctggaacg
agacagaccccgagttcgtgatcgaccacatggacgccaacaaggccggcagataccagtgtcagtaccggatcggccactaccggttcagatacagcgacaccctggaactggtggt
caccggcctgtacggcaagccttttctgtctgccgatagaggactggtgctgatgcccggcgagaacatcagcctgacctgtagctctgctcacatcccctcgacagattcagcctggcca
aagaaggcgagctgagcctgcctcagcatcagtctggcgaacaccccgccaacttttctctgggccctgtggacctgaacgtgtccggcatctacagatgctacggctggtacaatcgga
gcccctacctgtggtctttccccagcaatgccctcgaactggtcgtgaccgatagcatccaccaggactacaccacacagaacctgatcagaatggccgtggccggcctggttctggttgc
tctgctggctattctggtggaaaactggcacagccacacagccctgaacaaagaggcttctgccgacgtcgccgagccttcttggagtcagcagatgtgtcagcccggcctgaccttcgcc
agaacacctagcgtgtgcaag

SEQ 17:

Amino acid sequence of chimeric CD89 by exchanging EC1 part from human CD89 (Gly72 – Gly96) with EC1 part from cynomolgus monkey CD89 (**Asp72 – Gly96**)

MDPKQTTLLCLVLCLGQRIQAQEGDFPMPFISAKSSPVIPLDGSVKIQCQAIREAYLTQLMIIKNSTYREIDEKLGFWNDTTPEFVIDHMDANKAGRY
QCQYRIGHYRFRYSDTLELVVTGLYGKPFLSADRGLVLMPGENISLTCSSAHIPFDRFSLAKEGELSLPQHQSGEHPANFSLGPVDLNVSGIYRCYGW
YNRSPYLWSFPSNALELVVTDSIHQDYTTQNLIRMAVAGLVLVALLAILVENWHSHTALNKEASADVAEPSWSQQMCQPGLTFARTPSVCK

Signal peptide from human CD89 (aa sequence 1-21), chimeric human/cynomolgus monkey CD89 extracellular domain (aa sequence 22-227), comprising of an Ig-like EC1 domain part from human CD89 (aa sequence 22-71; Swiss-Prot no. P24071.1), an Ig-like EC1 domain part from cynomolgus monkey CD89 (aa sequence 72-96; NCBI Reference Sequence: XP_005590398.1) and an Ig-like EC1 domain part from human CD89 (aa sequence 97-121; Swiss-Prot no. P24071.1), of a short hinge region from human CD89 (aa sequence 122-125), of an Ig-like EC2 domain from human CD89 (aa sequence 126-220), and of a membrane proximal 'linker' region from human CD89 (aa sequence 221 -227), followed by transmembrane domain from human CD89 (aa sequence 228-246), and short cytoplasmic tail from human CD89 (aa sequence 247-287) according to Ding et al. J Biol Chem 2003; 278:27966-27970.

SEQ 18:

cDNA sequence coding for chimeric CD89 by exchanging EC1 part from human CD89 (Gly72 – Gly96) with EC1 part from cynomolgus monkey CD89 (**Asp72 – Gly96**) (optimized for mammalian expression)

atggaccccaagcagaccacactgctgtgcctggtgctgtgtctcggccagagaatccaagctcaagagggcgacttccccatgcctttcatcagcgccaagagcagccctgtgatccct
ctggatggcagcgtgaagatccagtgccaggccatcagagaggcctacctgacacagctgatgatcattaagaacagcacctaccgcgagatcgacgagaagctcggcttctggaacg

acaccacacctgagttcgtgatcgaccacatggacgccaacaaggccggcagataccagtgtcagtaccggatcggccactaccggttcagatacagcgacaccctggaactggtggtc
accggcctgtacggcaagccttttctgtctgccgatagaggactggtgctgatgcccggcgagaacatcagcctgacctgtagctctgctcacatcccсttcgacagattcagcctggcca
aagaaggcgagctgagcctgcctcagcatcagtctggcgaacaccccgccaacttttctctgggccctgtggacctgaacgtgtccggcatctacagatgctacggctggtacaatcgga
gcccctacctgtggtctttccccagcaatgccctcgaactggtcgtgaccgatagcatccaccaggactacaccacacagaacctgatcagaatggccgtggccggcctggttctggttgc
tctgctggctattctggtggaaaactggcacagccacacagccctgaacaaagaggcttctgccgacgtcgccgagccttcttggagtcagcagatgtgtcagcccggcctgaccttcgcc
agaacacctagcgtgtgcaag

SEQ 19:

Amino acid sequence of chimeric CD89 by exchanging EC1 part from human CD89 (Arg97– Gly121) with EC1 part from cynomolgus monkey CD89 (**Arg97– Gly121**)

MDPKQTTLLCLVLCLGQRIQAQEGDFPMPFISAKSSPVIPLDGSVKIQCQAIREAYLTQLMIIKNSTYREIGRRLKFWNETDPEFVIDHMDANKAGRY
RCRYRIGLSRFRYSDTLELVVTGLYGKPFLSADRGLVLMPGENISLTCSSAHIPFDRFSLAKEGELSLPQHQSGEHPANFSLGPVDLNVSGIYRCYGWY
NRSPYLWSFPSNALELVVTDSIHQDYTTQNLIRMAVAGLVLVALLAILVENWHSHTALNKEASADVAEPSWSQQMCQPGLTFARTPSVCK

Signal peptide from human CD89 (aa sequence 1-21), chimeric human/cynomolgus monkey CD89 extracellular domain (aa sequence 22-227), comprising of an Ig-like EC1 domain part from human CD89 (aa sequence 22-96; Swiss-Prot no. P24071.1) and an Ig-like EC1 domain part from cynomolgus monkey CD89 (aa sequence 97-121; NCBI Reference Sequence: XP_005590398.1), of a short hinge region from human CD89 (aa sequence 122-125), of an Ig-like EC2 domain from human CD89 (aa sequence 126-220), and of a membrane proximal 'linker' region from human CD89 (aa sequence 221 -227), followed by transmembrane domain from human CD89 (aa sequence 228-246), and short cytoplasmic tail from human CD89 (aa sequence 247-287) according to Ding et al. J Biol Chem 2003; 278:27966-27970.

SEQ 20: cDNA sequence coding for chimeric CD89 by exchanging EC1 part from human CD89 (Arg97– Gly121) with EC1 part from cynomolgus monkey CD89 (**Arg97– Gly121**) (optimized for mammalian expression)

atggaccccaagcagaccacactgctgtgcctggtgctgtgtctcggccagagaatccaagctcaagagggcgacttccccatgcctttcatcagcgccaagagcagccctgtgatccct
ctggatggcagcgtgaagatccagtgccaggccatcagagaggcctacctgacacagctgatgatcattaagaacagcacctaccgcgagatcggcagacggctgaagttctggaacg
agacagaccccgagttcgtgatcgaccacatggacgccaacaaggccggcagataccggtgcagatacagaatcggcctgagccggttccggtacagcgatacactggaactggtggt
caccggcctgtacggcaagccttttctgagcgccgatagaggactggtgctgatgcccggcgagaacatcagcctgacctgtagctctgctcacatccccttcgacagattcagcctggcc
aaagaaggcgagctgagcctgcctcagcatcagtctggcgaacaccccgccaacttttctctgggccctgtggacctgaacgtgtccggcatctacagatgctacggctggtacaatcgg
agcccctacctgtggtctttccccagcaatgccctggaactcgtcgtgaccgatagcatccaccaggactacaccacacagaacctgatcagaatggccgtggccggcctggttctggttg
ctctgctggctattctggtggaaaactggcacagccacacagccctgaacaaagaggcttctgccgacgtcgccgagccttcttggagtcagcagatgtgtcagcccggcctgaccttcgc
cagaacacctagcgtgtgcaag

S3. CD89 expression on tonsillar neutrophilic granulocytes.

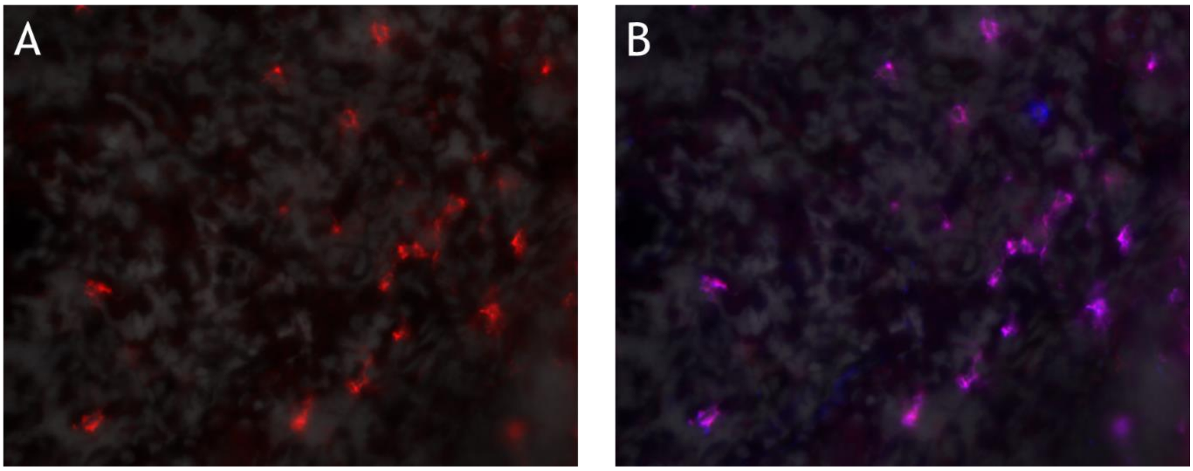

Single fluorescence microscopic image of tonsil demonstrating CD89 expression using mouse anti-CD89 mAb clone 10E7 (A). Overlay of fluorescence microscopic images of tonsil demonstrating CD89 expression using mouse anti-CD89 mAb clone 10E7 and neutrophil-specific marker CD66b using mouse anti-CD66B mAb (B). Grey represents nuclei (DAPI), red represents single mouse anti-CD89 mAb clone 10E7 staining, and purple represents double staining of CD89+ (red)/CD66b+ (blue) cells.

S4. CD89 staining on skin and pituitary

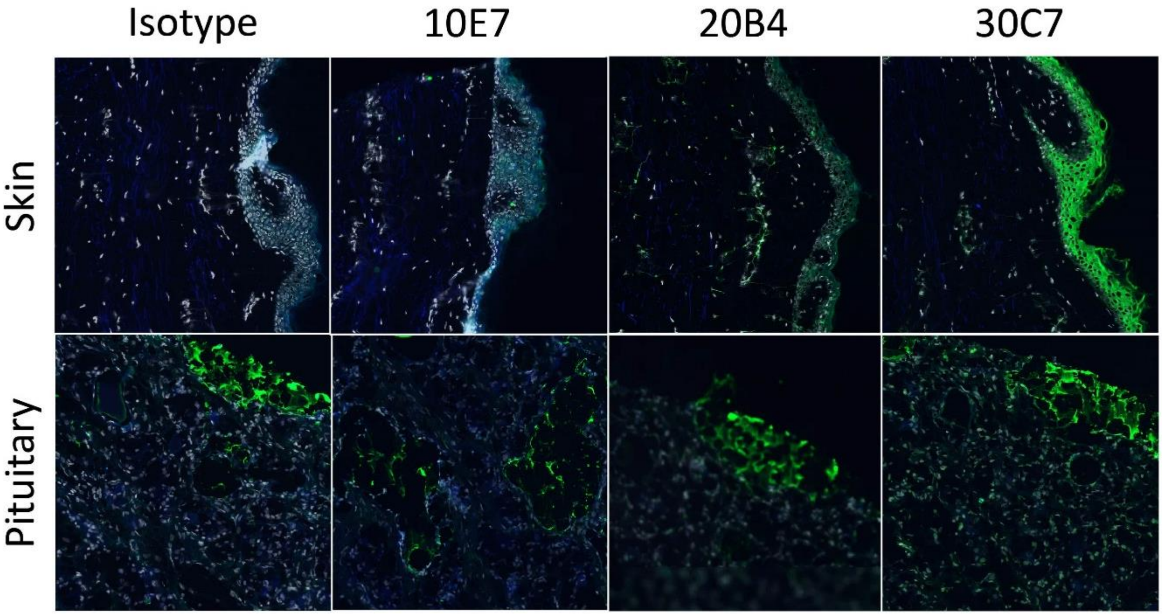

Staining of anti-CD89 clones on skin and pituitary. Some vague aspecific background staining was observed in the epidermis for clone 10E7; however this was also seen for the isotype control. Staining in the pituitary might be debris, and no co-localization with nuclei was observed. DAPI (grey; nucleus), CD66b (blue), CD89 (green), CD66b+CD89 (light green).
